# Right but Still Lousy: Correct Responses With an Unfavorable Outcome Elicit an Error Positivity

**DOI:** 10.1111/psyp.70151

**Published:** 2025-09-19

**Authors:** Peter Löschner, Marco Steinhauser

**Affiliations:** ^1^ Department of Psychology Catholic University of Eichstätt‐Ingolstadt Eichstätt Bavaria Germany

**Keywords:** error monitoring, error positivity, error‐related negativity, event‐related potentials

## Abstract

The error positivity (Pe) is a neural correlate of performance monitoring that is observed after errors in choice tasks. The results of previous studies suggest that the Pe reflects a monitoring process that goes beyond the mere distinction between correct and incorrect responses. Here, we investigated the idea that the Pe represents a higher‐order error signal reflecting an inference‐based outcome evaluation. To this end, we created a multistage task whose overall outcome depended on the correctness of each individual stage and was revealed not until the last stage. This implied that the final response could lead to an unfavorable outcome even if it was objectively correct. Our results replicated the general finding that a Pe occurs immediately after errors within each stage. Crucially, we also obtained a Pe after correct responses associated with an unfavorable outcome at the final stage. Moreover, a pattern classifier trained to decode this higher‐order Pe successfully decoded the Pe for incorrect responses. These results suggest that the Pe represents an evaluative process that infers the outcome by integrating multiple error signals and taking context into account.

## IMPACT STATEMENT

The error positivity (Pe) is an event‐related potential following errors in choice tasks that has previously been linked to error awareness and error confidence. Here, we demonstrate that a Pe is also observable after correct responses if these are associated with an unfavorable outcome. This substantially extends previous literature showing that the Pe reflects a higher‐order error signal resulting from an inference‐based outcome evaluation taking context into account.

## 1 Introduction

To efficiently adapt behavior, monitoring processes are required that detect negative outcomes and adjust behavior accordingly. In experimental tasks with a single behavioral response, this monitoring mainly involves distinguishing between correct and incorrect responses. In such scenarios, event‐related potentials (ERPs) such as the error‐related negativity (Ne/ERN; Falkenstein et al. 1991; Gehring et al. 1993) and the error positivity (Pe; Overbeek et al. 2005) are studied as they represent distinct processes of error monitoring. However, everyday tasks often consist of several behavioral responses distributed over multiple stages, and the overall outcome of such a multistage task can be determined not until the last stage is finished. In such a scenario, not only the correctness of single responses but the outcome of the whole multistage task must be evaluated. Here, we hypothesize that the Pe represents a higher‐order error signal that indicates the overall outcome in a multistage task. To investigate this, we created a multistage task in which the overall outcome can be unfavorable even if the last response is correct and tested whether this response leads to a Pe.

The Pe is a slow parietal positive deflection that peaks between 200 and 400 ms post‐response (Falkenstein et al. 1991; Overbeek et al. 2005). It has been linked to error awareness (Endrass et al. 2007; Nieuwenhuis et al. 2001; O'Connell et al. 2007), leading to the theory that the Pe reflects the accumulation of evidence that an error has been made (Steinhauser and Yeung 2010, 2012; Ullsperger et al. 2010). The Pe has also been associated with decision confidence (Boldt and Yeung 2015), motivational processes (Drizinsky et al. 2016; Schroder et al. 2014) or negative affect (Falkenstein et al. 2000; Rodeback et al. 2020). In contrast, the Ne/ERN is an earlier fronto‐central negativity observed immediately following erroneous responses, peaking between 50 and 100 ms post‐response (Falkenstein et al. 2000; Ullsperger, Danielmeier, and Jocham 2014). Theoretical accounts suggest that the Ne/ERN reflects a mismatch between the given and actually correct response (Coles et al. 2001), a post‐response conflict (Yeung et al. 2004) or a prediction error (Holroyd and Coles 2002). In each of these accounts, the Ne/ERN represents a rapid error signal indicating that the immediately preceding response is incorrect.

While the Pe and Ne/ERN have initially been interpreted as reflecting different stages of the same error monitoring process, there is increasing evidence that they represent dissociable monitoring systems that evaluate behavior in different ways. Di Gregorio et al. (2018) showed that, in a situation in which participants can infer that an error has occurred without knowing the correct response, a Pe can occur in the absence of an Ne/ERN (see also Hewig et al. 2011). Their results suggest that, while the Ne/ERN relies on a comparison of correct and actual responses, the Pe represents an inference‐based evaluation that takes context information into account. In another study (Pfister et al. 2016) participants could intentionally commit errors by breaking task rules. These intentional errors did not elicit an Ne/ERN but a Pe‐like positivity, which might reflect that intentional errors are interpreted as a negative outcome (resulting in a Pe) even though they are not incorrect as they do not deviate from an intention (resulting in no Ne/ERN). Finally, dual‐tasking experiments revealed that a Pe can even occur after a correct response (Steinhauser and Steinhauser 2021). When two tasks were performed in close succession, an error in the first task elicited a Pe following the correct response in the second task. This was interpreted as reflecting a temporal deferment of error monitoring if both tasks overlap in time.

The results described above suggest that the Pe represents an outcome evaluation that goes beyond the mere distinction between correct and incorrect as reflected by the Ne/ERN. Here, we propose that the Pe represents a higher‐order error signal that is based on an inference‐based outcome evaluation which takes context information into account. To investigate this idea, we consider a multistage task that consists of a series of decisions, but whose overall outcome depends on the correctness of each individual stage and is revealed not until the last stage. Such a task requires error monitoring on two hierarchical levels: the level of individual responses and the level of the overall outcome. Crucially, we assume that the Ne/ERN indicates error monitoring on the response level, whereas the Pe represents a higher‐order signal that tracks the overall outcome. To test this, we created a situation within our multistage task in which the response at the last stage is correct, whereas the overall outcome is suboptimal. For this situation, we predicted that the correct response will elicit a Pe (but no Ne/ERN) indicating that the overall outcome is unfavorable. Such a result would not only demonstrate that the Pe relates to a higher‐order error signal reflecting the outcome of a task (rather than the correctness of a response), it would provide further evidence for a dissociation between Ne/ERN and Pe (Di Gregorio et al. 2018).

### 1.1 The Present Study

**FIGURE 1:**
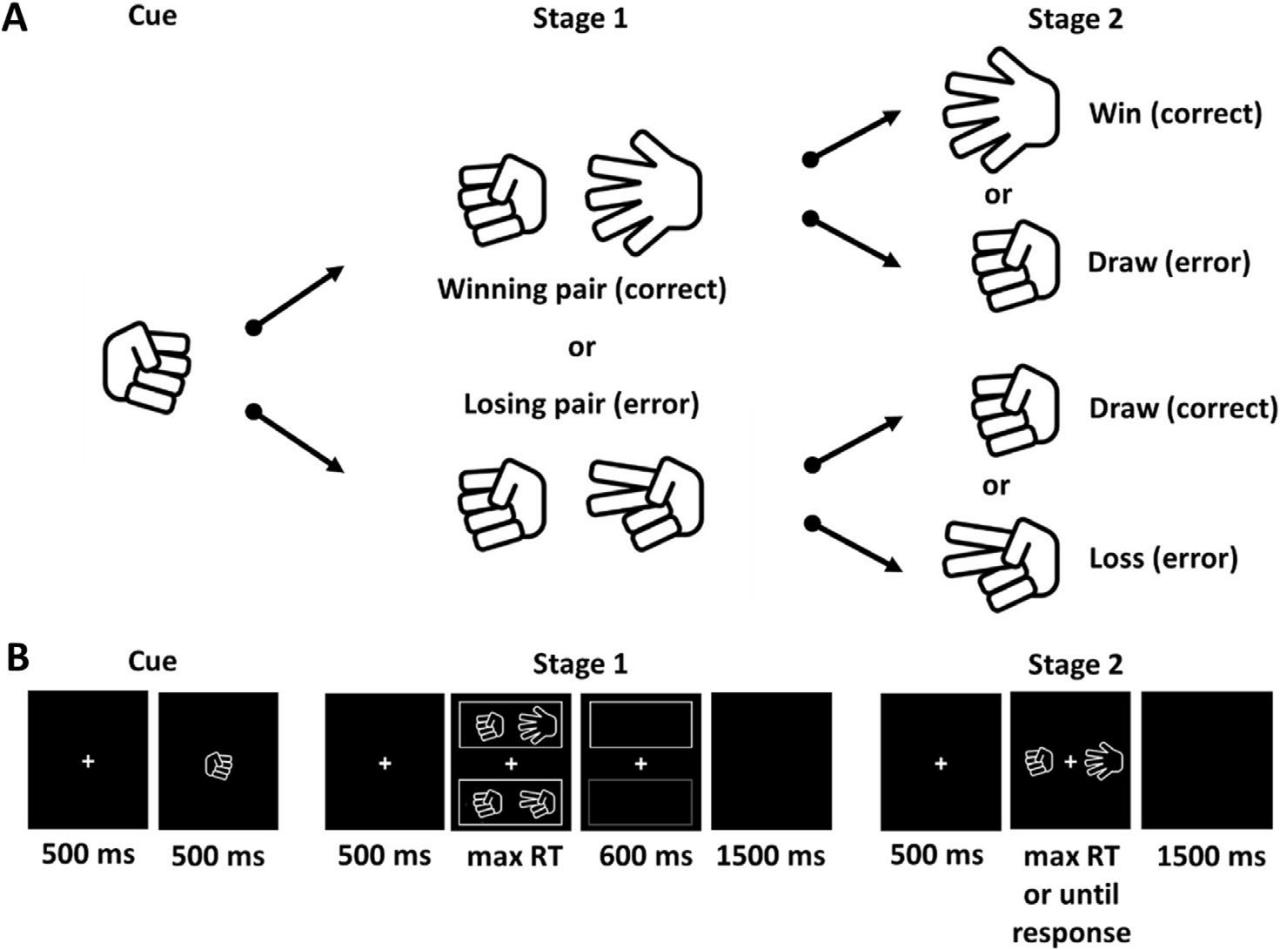
Overview of an exemplary trial. (A) All possible decisions within the trial. (B) Time course of the trial. First, a cue is shown indicating the gesture of the opponent (here the rock). On Stage 1, participants have to choose between the winning pair (containing the cue gesture and the winning gesture, here the rock and the paper) and the losing pair (containing the cue gesture and the losing gesture, here the rock and the scissor). On Stage 2, participants have to choose from the two gestures of the pair chosen in Stage 1. Participants can make a correct response or an error at Stage 2 leading to an overall win, a loss, or a draw. max RT = maximum response time of the respective stage.

In the present study, we created an experimental paradigm inspired by the game “Rock, Paper, Scissors”. In the original game, players repeatedly form one of three hand gestures representing a rock, a paper, and a scissor. To determine which player has won the round, the three gestures establish an intransitive set of relations with rock winning against scissor, scissor winning against paper, and paper winning against rock. If both players show the same gestures, the round is counted as draw. In our version, this game is turned into a two‐stage decision in which the final outcome depended on both decision stages (Figure 1B). Initially, participants are shown the gesture they are playing against (the cue, e.g., the rock). At Stage 1, participants have to choose from two pairs of gestures: The winning pair contains the cue together with a winning gesture (e.g., the rock and the paper). The losing pair contains the cue together with a losing gesture (e.g., the rock and the scissor). At Stage 2, participants have to choose a gesture from the previously chosen Stage‐1 pair. The final outcome depends on whether the chosen gesture wins or loses against the cue gesture, or whether cue and chosen gesture are the same (draw).

The structure of this two‐stage task implies that the final outcome depends on the decisions at both stages (Figure 1A): If the winning pair was chosen at Stage 1, the final outcome can be either a win or a draw. But if the losing pair was chosen at Stage 1, the final outcome can be a draw or a loss. To maximize the outcome, the *correct* choices would be the winning pair at Stage 1 and the winning gesture (if choosing from a winning pair) or the gesture resulting in a draw (if choosing from a losing pair) at Stage 2. As we were interested in deviations from optimal outcomes; however, we enforced *errors* by inducing speed pressure. An error occurred if the losing pair was chosen at Stage 1, or if the losing gesture (if choosing from a losing pair) or the gesture resulting in a draw (if choosing from a winning pair) was chosen at Stage 2. In either of these cases, we expected to observe markers of error monitoring such as the Ne/ERN and Pe after these incorrect choices. However, crucial for the present purpose is the scenario in which a Stage‐1 error is followed by a correct response at Stage 2. In this case, the losing pair is chosen at Stage 1, which implies that the best possible final outcome at Stage 2 is a draw. This creates a situation in which a correct Stage‐2 response leads to an unfavorable outcome. The question is whether the Stage‐2 response in this situation is associated with a Pe. Such a finding would demonstrate that the Pe reflects a higher‐order error signal that indicates unfavorable outcomes even if the response was correct.

## 2 Method

### 2.1 Participants

A priori power analysis revealed that a sample size of 24 participants was required to allow the crucial within‐subjects t‐test testing for the presence of a Ne/ERN or Pe to detect medium effect sizes of 0.52 with a statistical power of 0.8 (G*Power; Faul et al. 2007, 2009). After initial data from 24 participants were collected, one participant had to be excluded because no trials remained for analyzing Stage‐2 errors after EEG preprocessing. Another participant had to be excluded because less than 6 trials remained for analyzing higher‐order errors (Pontifex et al. 2010). These two were replaced with new participants. This resulted in a sample of 24 usable participants (4 male, 0 diverse; age *M* = 23.3 years, SD = 4.17; all right‐handed). All participants were recruited at the Catholic University of Eichstätt‐Ingolstadt and received either course credit or payment (10.50 € per hour) in addition to a performance‐dependent monetary incentive of up to 4.80 €. All participants gave informed consent, and the study was approved by the ethics committee of the Catholic University of Eichstätt‐Ingolstadt.

### 2.2 Stimuli

Stimuli were three hand gestures representing the options of the game “rock, paper, scissors,” which were either presented as pairs of options with the fingers on the left (Stage 1 in Figure 1) or as cues with the fingers on the right (Cue in Figure 1). On the screen, the rock gesture had a height of 1.30° and a width of 1.22°, the paper gesture had a height of 1.72° and a width of 1.60°, and the scissor gesture had a height of 1.30° and a width of 1.60°. All gestures were drawn with white lines on a black background. Stimuli were presented on an Acer (XB253Q) flatscreen with a frame rate of 144 Hz.

### 2.3 Task and Procedure

Each trial consisted of two stages and each stage required a decision by the participants (Figure 1). The trial started with a central fixation cross displayed for 500 ms. This was followed by a gesture representing the cue for 500 ms. After another fixation cross for 500 ms, the first‐stage stimulus appeared, which consisted of two pairs of gestures, each within a white frame above and below the fixation cross. One of the pairs was the same gesture as the cue together with the gesture that would win against the cue (the winning pair). The other pair was the same gesture as the cue together with the gesture that would lose against the cue (the losing pair). The position of gestures (left, right) as well as pairs (up, down) was random and balanced within each block. Participants had to make their Stage‐1 decision by pressing the arrow‐up or arrow‐down key with the index finger of their dominant hand. After the response, only the two frames were shown for 600 ms (whereas the gestures disappeared) and the frame not chosen was darkened. Then, a black screen was presented for 1500 ms. Stage 2 started with a fixation cross for 500 ms followed by the pair of gestures chosen in Stage 1 presented left and right of a fixation cross. The position of the two gestures was randomized. Participants made their Stage‐2 decision by pressing the arrow‐left or arrow‐right key with the same finger as for the Stage‐1 decision. After the response, a black screen was depicted for 1500 ms until the next trial started. Participants were instructed to respond as quickly and as accurately as possible and to relocate their index finger in the middle of the arrow keys after each response.

To ensure an adequate error rate, adaptive response windows were employed at each stage to induce time pressure. If a maximum response time (RT) has passed without a response, a feedback screen (“Zu spät!”, engl. too late) was presented immediately for 1000 ms (and no frames were depicted at Stage 1). If participants pressed a key prior to the presentation of a stimulus at any stage, feedback was provided for a duration of 1000 ms (“Zu früh gedrückt!”, engl. pressed too early) and the next trial started. The maximum RT was adjusted separately for each stage and depended on the error rate of the preceding 20 trials at this stage. The targeted error rate ranged between 15% and 20% for Stage 1 and between 5% to 10% for Stage 2. If the error rate fell below 15% at Stage 1 or 5% at Stage 2, the maximum RT for the respective stage was decreased by 30 ms. If the error rate exceeded 20% at Stage 1 or 10% at Stage 2, the maximum RT for the respective stage was increased by 30 ms. If a response was too late, then the maximum RT was additionally increased by 60 ms. The starting value for each stage was 1500 ms. The maximum RT could not exceed 1500 ms. Additionally, to prevent too many too late responses, the lower limit for the maximum RT was the 90th percentile of the participant's RT of all preceding trials. This adaptive response window led to a maximum RT of 1053 ms (SE = 56 ms) for Stage 1 and 950 ms (SE = 56 ms) for Stage 2.

To incentivize correct and quick responding, a monetary incentive system was implemented. In each trial, participants received points based on their final outcome, and the score of accumulated points was exchanged for money at the end of the experiment (10 points = 1 ct.). Ten points were added for a win, and five points were subtracted for a loss. No points were added or subtracted for a draw. A failure to respond within the maximum RT led to an additional loss of 30 points. The current score was displayed for 4000 ms after every 12th trial, followed by a black screen for 500 ms before the next trial started.

The experiment comprised 10 test blocks with 48 trials each, resulting in a total of 480 trials. Oral as well as written feedback emphasizing the need to respond either faster or more accurately was administered after each block. Feedback was triggered if the error rate exceeded predefined thresholds set at 15%–20% for Stage 1 and 5%–10% for Stage 2. Prior to these test blocks, participants conducted four practice blocks to familiarize participants with the task and the monetary incentive system. The first practice block comprised 12 trials in which only Stage 2 without a response window was practiced. The second practice block comprised 24 trials with both stages but again without response windows. The third and fourth practice blocks comprised 48 trials, in which the response windows and the incentives were introduced. After the Stage‐2 response, participants received immediate feedback on the awarded points in the current trials (“10”, “0”, “‐5”, or “−30”), which was presented 1000 ms after the response for 1000 ms, followed by a black screen for 500 ms. After these practice blocks, participants were informed that, in the test blocks, there was no trial‐wise feedback about the outcome but that a score was presented every 12 trials. This score was reset to zero at the beginning of the test blocks.

### 2.4 EEG Data Acquisition

64 Ag‐AgCl electrodes were used in a 10‐10 system to acquire EEG‐Data with a BioSemi Active‐Two system (BioSemi, Amsterdam, The Netherlands; channels Fp1, AF7, AF3, F1, F3, F5, F7, FT7, FC5, FC3, FC1, C1, C3, C5, T7, TP7, CP5, CP3, CP1, P1, P3, P5, P7, P9, PO7, PO3, O1, Iz, Oz, POz, Pz, CPz, Fpz, Fp2, AF8, AF4, AFz, Fz, F2, F4, F6, F8, FT8, FC6, FC4, FC2, FCz, Cz, C2, C4, C6, T8, TP8, CP6, CP4, CP2, P2, P4, P6, P8, P10, PO8, PO4, O2 and the left and right mastoid). For reference and ground electrodes, the Common Mode Sense (CMS) and Driven Right Leg (DRL) electrodes were used, which were placed centrally and close to POz. Additionally, the vertical and horizontal electrooculogram (EOG) was recorded with BioSemi FLAT Active electrodes (Ag‐AgCl) above and below the right eye and on the outer canthi of both eyes. Both EEG and EOG were recorded at a sampling rate of 1024 Hz, and no online filtering was applied. All electrodes were off‐line re‐referenced to averaged mastoids.

### 2.5 Data Analysis

#### 2.5.1 Behavioral Data Analysis

Each response was assigned to the condition Stage (Stage 1, Stage 2) and classified as a correct response or error. At Stage 1, the response was correct if the winning pair was chosen and an error if the losing pair was chosen. At Stage 2, the response was correct if the winning gesture (if choosing from a winning pair) or the gesture that results in a draw (if choosing from a losing pair) was chosen. An error occurred at Stage 2 if the losing gesture (if choosing from a losing pair) or the gesture resulting in a draw (if choosing from a winning pair) was chosen. A correct Stage‐2 response that was preceded by a Stage‐1 error was defined as a higher‐order error. All trials were excluded whose RTs deviated more than three standard deviations from the RT mean of each condition and participant. Furthermore, trials featuring responses either too early (*M* = 0.31%, SE = 0.10%) or too late (*M* = 4.97%, SE = 0.45%) were excluded from subsequent analysis. Prior to statistical testing of error rates, arcsine transformations were applied (Winer et al. 1991). We first compared error rates of the two stages using a paired t‐test. Then, RTs were subjected to a two‐way repeated measures ANOVA with the variables Stage (Stage 1 vs. Stage 2) and Correctness (correct vs. error). To address post‐error adjustments, the error rate at Stage 2 and the RTs of correct Stage‐2 responses were compared between Stage‐2 responses following a correct Stage‐1 response and those following a Stage‐1 error using paired t‐tests.

#### 2.5.2 EEG Preprocessing

All analyses were conducted using custom scripts in MATLAB v9.10 (The Mathworks, Natick, MA) and EEGLAB v2019.1 (Delorme and Makeig 2004). Continuous EEG data was downsampled to 512 Hz, low‐pass filtered (40 Hz) and then high‐pass filtered (0.1 Hz) with a least‐squares FIR filter (−6 dB half‐amplitude cutoff). Epochs ranging from −500 ms before to 1000 ms after each response (Stage 1 and Stage 2) were extracted and baseline‐corrected using a baseline interval from −150 to −50 ms before the response. Bad electrodes were identified using EEGLAB's channel rejection routine (pop_rejchan.m) with a joint probability and a kurtosis criterion (both with a threshold of 5) and were interpolated using spherical spline interpolation. On average, 4.39% of electrodes for Stage 1 (SE = 0.62%) and 3.79% for Stage 2 (SE = 0.56%) were interpolated. To correct eye blinks and muscular artifacts, an infomax‐based Independent Component Analysis (Bell and Sejnowski 1995) was conducted on a copy of the dataset to which an additional 1 Hz high‐pass filter was applied. Independent components (ICs) reflecting ocular and motor artifacts were then identified using the ICLabel toolbox (Pion‐Tonachini et al. 2019) and removed from the original dataset. On average, 10.0 ICs were removed per participant for Stage 1 (SE = 0.86) and 11.92 for Stage 2 (SE = 1.03). Of these, a mean of 4.46 ICs in Stage 1 (SE = 0.73) and 4.33 in Stage 2 (SE = 0.70) represented motor noise which might reflect that participants had to move their fingers to the respective keys. Retaining these ICs would have led to a more than two times higher number of excluded trials in the next preprocessing step. In this next step, epochs whose channel activity exceeded +/−100 μV or whose joint probability deviated more than 5 standard deviations from the mean within the time range of interest (−150 to 400 ms) were removed. On average, 9.90% of trials in Stage 1 (SE = 1.54%) and 10.55% of trials for Stage 2 (SE = 2.21%) were removed in this way. Finally, epochs were averaged separately for Stage‐1 responses and Stage‐2 responses and separately for the different conditions as described below. No trials with errors in both stages were analyzed because these trials were too infrequent (*M* = 6.75, SE = 0.96). The number of trials in the remaining conditions ranged between 13.9 (SE = 1.99) for Stage‐2 errors following correct Stage‐1 responses and 332.6 (SE = 9.96) trials for correct Stage‐2 responses following correct Stage‐1 responses. Note that, in the former condition, four participants had less than 6 trials, which has been recommended as a minimum to analyze the Ne/ERN (Pontifex et al. 2010). Therefore, after the above‐described replacement of two participants, we additionally conducted all analyses described below without participants with less than 6 trials. These analyses replicated all effects from the full sample if not stated otherwise. Because the recommendation of Pontifex et al. (2010) is valid for the Ne/ERN while our component of interest was the Pe, we additionally applied the method of Luck et al. (2021) to check whether small trial numbers in some participants influenced our results. That is, we calculated standardized measurement errors (SME) for each component (Ne/ERN, Pe) and condition for the time windows used in the respective analyses. For Stage‐2 errors, SMEs would have been improved when one participant was excluded for the Ne/ERN and two participants were excluded for the Pe. When conducting our analyses (including the MVPA) without these participants, almost all results remained the same (with one exception noted in the results section). We therefore retained these participants in the reported analyses.

#### 2.5.3 ERP Data Analysis

The Pe and Ne/ERN were defined as the difference in mean amplitudes between errors and correct trials (for a discussion of this definition, see Discussion section). As participants had to move their finger to the respective response keys and ERP data were locked to the keypress rather than to the movement onset, we had to deal with a temporal offset in response‐locked ERP components. This was relevant mainly for the Ne/ERN but less so for the more long‐going Pe. We therefore did not predefine a time window for the Ne/ERN but used a cluster‐based permutation test within a broader time period at electrode FCz. In contrast, the Pe was quantified within the frequently used time window of 200 ms to 400 ms (Endrass et al. 2007; Overbeek et al. 2005; Ullsperger, Fischer, et al. 2014) at electrode POz (Steinhauser and Yeung 2012; Steinhauser and Steinhauser 2021). Please note that the Pe is often quantified at more anterior electrodes such as Pz or CPz. To make sure that choosing POz does not inappropriately bias our data, we applied all Pe analysis in the present study also for data at electrodes Pz and CPz but obtained the same results for all analysis.

First, we investigated whether a typical Ne/ERN and the Pe were present for Stage‐1 errors and Stage‐2 errors. Note that because trials with errors in both stages were excluded, Stage‐1 and Stage‐2 errors refer to trials with a correct response in the other stage. For the Ne/ERN, we used a cluster‐based permutation test to compare correct and incorrect responses at each stage. Using the Mass Univariate ERP Toolbox (Groppe et al. 2011), we identified clusters and calculated 100,000 permutations within the time range of −50 to 200 ms at FCz. A cluster inclusion threshold as well as an output threshold of *p* = 0.05 was used. For the Pe, we performed within‐subject t‐tests comparing errors with correct trials at each stage. We then analyzed both components in higher‐order errors by comparing correct Stage‐2 responses following Stage‐1 errors with those following correct Stage‐1 responses using the same methods as described above. We finally compared all three error types (Stage‐1 error, Stage‐2 errors, higher‐order errors) by subjecting Ne/ERN and Pe amplitudes to a one‐way repeated measures ANOVA with error type as the variable. Pairwise differences were tested using paired t‐tests. Ne/ERN amplitudes were quantified in the time windows identified by the permutation tests in the previous step. As no significant cluster was found for higher‐order errors, the time window for Stage‐2 errors was used.

#### 2.5.4 Multivariate Pattern Analysis (MVPA)

An MVPA was used to investigate whether the error‐related brain activity found for higher‐order errors shows a similar spatial distribution as that for Stage‐2 errors. To this end, we used a rationale that has been applied in previous studies (Boldt and Yeung 2015; Di Gregorio et al. 2018; Steinhauser and Yeung 2010, 2012). First, a logistic regression classifier was trained to discriminate between correct Stage‐2 responses following a Stage‐1 error (corresponding to a high‐order error) and correct Stage‐2 responses following correct Stage‐1 responses using the linear integration method (Parra et al. 2002, 2005). Classifier input was the mean single‐trial amplitude from all 64 electrodes in a specific time window. To trace the time course of classifier performance, classifiers were trained for consecutive but overlapping time windows in the interval between −100 and 500 ms with a width of 50 ms and a step size of 25 ms. The training set comprised all correct Stage‐2 responses following a Stage‐1 error (the higher‐order error trials) as well as half of the correct Stage‐2 responses following correct Stage‐1 responses (the all‐correct trials) as the other half was used for a later analysis step. For this, we divided the correct Stage‐1 responses into trials with odd and even trial numbers and used trials with odd numbers for training. To balance the number of higher‐order errors and all‐correct trials, the set of higher‐order error trials (which were less frequent in all participants) was duplicated until the difference in trial numbers between the two conditions was smaller than the number of higher‐order trials. Then, higher‐order trials were randomly drawn and added to the training set until trial numbers in both conditions were balanced. A leave‐one‐out cross‐validation procedure was used to evaluate the classifiers' performance. Each trial was classified after training with a training set from which this trial (and its duplicates) had been removed. The removed trials were replaced using a similar method as described in the previous step.

Classifier performance was measured by an Az score with a value of 0.5 indicating classification on a chance level and a value of 1 indicating perfect classification. A permutation test was used to test whether Az scores differed from chance level. For each participant, we randomized the assignment of trials to the two categories (higher‐order error vs. all correct) and calculated an Az score. Repeating this procedure 1000 times resulted in a distribution of Az scores, and the 5%‐percentile from this distribution was used as a critical Az score. Inference testing for each classifier was done by comparing the mean Az score (averaged across participants) to the respective mean critical Az score. To visualize the spatial distribution of the discriminating component, we provide a topography of coupling coefficients representing the activity at each electrode site that correlates with the discriminating component (Parra et al. 2002, 2005).

As the time range in which our classifiers showed significant classification performance strongly overlaps with the time period of the Pe, we averaged across all significant classifiers and concluded that the resulting classifier can be viewed as a decoder of the Pe‐like component elicited by higher‐order errors (which was also confirmed by the topography of the discriminating component). In a second step, we then tested whether this classifier can also distinguish between Stage‐2 errors and correct Stage‐2 responses in the Pe time range. This would provide evidence that similar ERP components (here: a Pe) are involved in both types of errors. To this end, we applied the classifier to Stage‐2 errors (following correct Stage‐1 responses) and those all‐correct trials that were not chosen for the training set (trials with even numbers). Prediction values were calculated for each trial and time point within the epoch. These prediction values can be interpreted as reflecting how strongly the spatial distribution of brain activity at this time point resembles the Pe‐like activity that is decoded by the classifier. Prediction values were averaged separately for Stage‐2 errors and correct Stage‐2 trials and compared on a group level using a paired t‐test.

## 3 Results

### 3.1 Behavioral Data

Error rates and RTs for Stage 1 and Stage 2 are depicted in Table 1. Participants made more errors in Stage 1 than in Stage 2, *t*(23) = 43.06, *p* < 0.001, d = 0.65, which is in accordance with the adaptive RT criterion aiming for more errors within Stage 1. The analysis of RTs revealed a significant main effect for Stage, *F*(1,23) = 32.26, *p* < 0.001, $\eta_{p}^{2}$ = 0.58, but no significant effect of Correctness, *F*(1,23) = 0.22, *p* = 0.641, $\eta_{p}^{2}$ < 0.01, and no significant interaction, *F*(1,23) = 2.60, *p* = 0.121, $\eta_{p}^{2}$ = 0.10. These results indicate faster RTs for Stage 2 (*M* = 446 ms, SE = 12 ms) than for Stage 1 (*M* = 502 ms, SE = 14 ms), again reflecting differences in the adaptive RT criterions. We next investigated whether Stage‐2 performance was affected by the correctness of the Stage‐1 response (Table 1). Participants made significantly more errors in Stage 2 after an erroneous Stage‐1 response than after a correct Stage‐1 response, *t*(23) = 34.34, *p* < 0.001, d = 0.60. However, Stage‐2 RT was not affected by the correctness of the Stage‐1 response, *t*(23) = 1.37, *p* = 0.183, d = 0.57, indicating no post‐error slowing.

**TABLE 1:** Error rates and response times for Stage 1 and Stage 2.

	Error rates (%)	Correct RT (ms)	Error RT (ms)
Stage 1	15.08 (1.15)	506 (6)	500 (10)
Stage 2	5.60 (1.5)	446 (7)	461 (10)
Stage 2 post correct	4.45 (1.40)	446 (7)	447 (6)
Stage 2 post error	14.05 (1.40)	470 (14)	450 (10)

*Note:* Within‐subjects standard errors (Morey 2008) are provided in parentheses.

Abbreviation: RT = response time.

### 3.2 ERP Data

**FIGURE 2:**
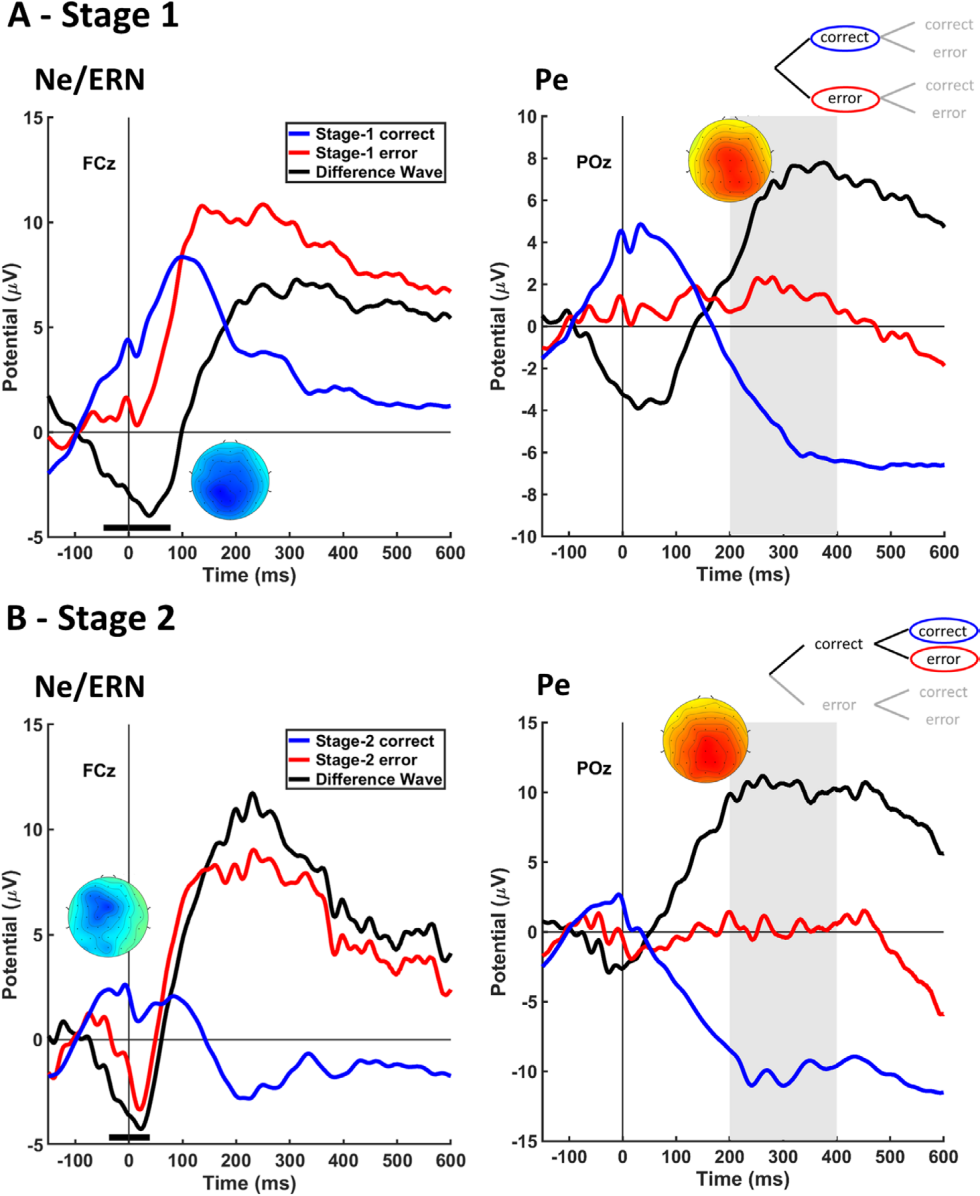
Error‐related brain activity for Stage‐1 errors and Stage‐2 errors. Panels show Stage‐1 locked (A) and Stage‐2 locked (B) ERPs and difference waves (errors minus corrects) at electrode FCz for analyzing the Ne/ERN and at POz for analyzing the Pe. Black horizontal lines indicate the cluster representing the Ne/ERN as revealed by the permutation test. Gray areas indicate the time interval for statistical testing of the Pe. Scalp topographies indicate the spatial distribution of the difference wave in the time interval representing the respective component.

Response‐locked ERPs used to analyze the Pe and Ne/ERN amplitudes for Stage 1 and Stage 2 are depicted in Figure 2. For Stage 1, we found the typical Pe pattern of a positive posterior deflection in errors (*M* = 1.55 μV, SE = 1.33 μV) relative to correct trials (*M* = −4.73 μV, SE = 1.35 μV) in the time range of 200–400 ms at electrode POz, *t*(23) = 5.52, *p* < 0.001, d = 0.94 (right panel in Figure 2A). For the Ne/ERN, the cluster‐based permutation revealed significantly more negative pronounced errors (*M* = 1.53 μV, SE = 0.65 μV) compared to correct trials (*M* = 4.52 μV, SE = 0.54 μV) in a time window of −47 to 78 ms (*p* < 0.001) at electrode FCz (left panel in Figure 2A). Unexpectedly, the spatial distribution of this Ne/ERN was not restricted to frontocentral electrodes but spanned across both anterior and posterior electrode sites.

For Stage 2, we also found an increased positivity for errors (*M* = 0.28 μV, SE = 1.60 μV) compared to correct trials (*M* = −9.98 μV, SE = 1.24 μV) at electrode POz indicating a Pe, *t*(23) = 8.40, *p* < 0.001, d = 1.44 (right panel in Figure 2B). For the Ne/ERN, a negative cluster in the time window of −37 to 39 ms (*p* = 0.030) was identified at electrode FCz which indicated a more negative activity for errors (*M* = −1.53 μV, SE = 0.90 μV) than for correct trials (*M* = 1.83 μV, SE = 0.70 μV). In contrast to Stage‐1 errors, the Ne/ERN for Stage‐2 errors had a clear frontocentral distribution.

**FIGURE 3:**
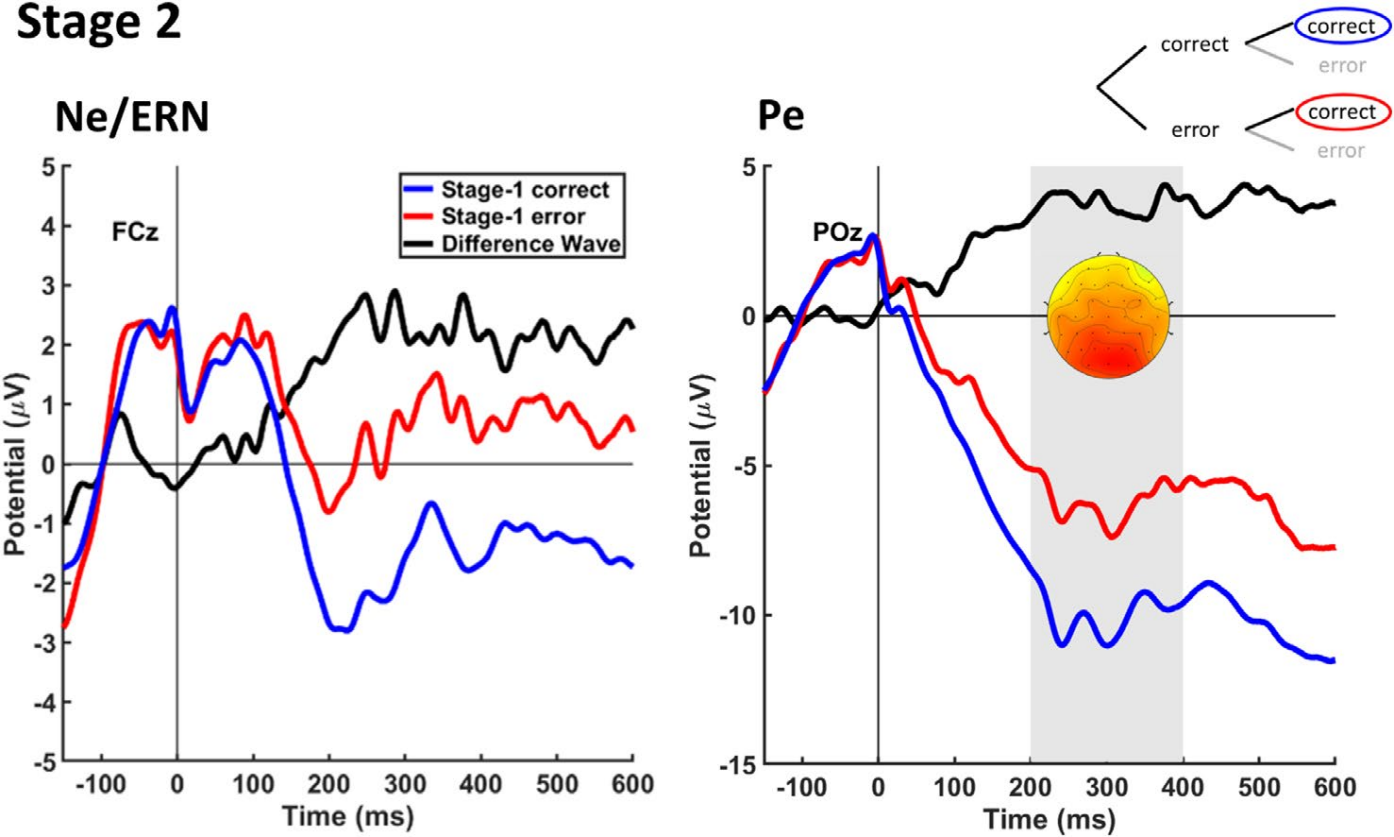
Error‐related brain activity reflecting higher‐order errors at Stage 2. Panels show Stage‐2‐locked ERPs and difference waves (error minus corrects) at electrode FCz for analyzing the Ne/ERN and at POz for analyzing the Pe. The gray area indicates the time interval for statistical testing of the Pe. The scalp topography indicates the spatial distribution of the difference wave in the time interval of the Pe.

After demonstrating that errors at each stage elicit the typical error‐related brain activity, we investigated whether also a correct Stage‐2 response leads to a Pe if this correct response was associated with an unfavorable outcome due to a Stage‐1 error. To this end, we compared Stage‐2 correct responses with a preceding Stage‐1 error with those with a preceding correct Stage‐1 response (see Figure 3). As hypothesized, we found such a higher‐order Pe as reflected by a more positive deflection in correct Stage‐2 responses following a Stage‐1 error (*M* = −6.20 μV, SE = 1.61 μV) than in those following a correct Stage‐1 response (*M* = −9.98 μV, SE = 1.24 μV) at electrode POz, *t*(23) = 4.85, *p* < 0.001, d = 0.53 (right panel in Figure 3). In contrast, the cluster‐based permutation test at electrode FCz did not find a negative cluster representing an Ne/ERN (left panel in Figure 3).

**FIGURE 4:**
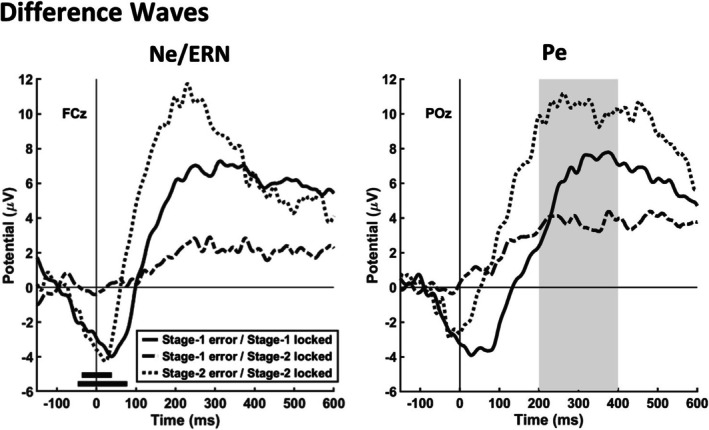
Difference waves (errors minus corrects) representing the Ne/ERN at electrode FCz and the Pe at electrode POz for Stage‐1 errors in Stage‐1‐locked data as well as Stage‐2 errors and higher‐order errors in Stage‐2‐locked data. The black horizontal lines indicate the time windows found by the permutation tests for the Ne/ERN. The gray area indicates the time interval for statistical testing for the Pe.

We then compared the three conditions (Stage‐1 errors, Stage‐2 errors, correct Stage‐2 responses following Stage‐1 errors) by considering the difference waves representing the respective Pes and Ne/ERNs (Figure 4). A one‐way ANOVA for the Pe revealed that the three conditions differed significantly, *F*(2, 46) = 16.22, *p* < 0.001, $\eta_{p}^{2}$ = 0.41. The Pe for Stage‐2 errors was significantly larger (*M* = 10.25 μV, SE = 1.22 μV) than the Pe for Stage‐1 errors (*M* = 6.28 μV, SE = 1.14 μV), *t*(23) = 4.00, *p* < 0.001, d = 1.06. Moreover, the higher‐order Pe (correct Stage‐2 responses following Stage‐1 errors) was significantly smaller (*M* = 3.77 μV, SE = 0.77 μV) compared to both, the Pe for Stage‐1 errors, *t*(23) = −2.11, *p* = 0.046, d = 0.52, and the Pe for Stage‐2 errors, *t*(23) = −5.20, *p* < 0.001, d = 1.27. Please note that the former comparison between higher‐order Pe and Pe for Stage‐1 errors did not reach significance, *t*(21) = −1.62, *p* = 0.120, d = 0.35, when the two participants with low SMEs were removed from the sample (see Section 2.5.2). To compare the Ne/ERN for the three conditions, we used the time windows associated with the respective clusters for Stage‐1 and Stage‐2 errors. For correct Stage‐2 responses following Stage‐1 errors, we used the time window associated with the cluster for Stage‐2 errors. The one‐way ANOVA again revealed that the three conditions differed significantly, *F*(2, 46) = 5.68, *p* = 0.013, $\eta_{p}^{2}$ = 0.12. We found no significant difference between Ne/ERNs for Stage‐1 errors (*M* = −2.99 μV, SE = 0.58 μV) and Stage‐2 errors (*M* = −3.36 μV, SE = 1.09 μV), *t*(23) = 0.38, *p* = 0.710, d = 0.16 (Figure 4). However, the Ne/ERN for correct Stage‐2 responses following Stage‐1 errors (*M* = −0.18 μV, SE = 0.68 μV) was significantly smaller than the Ne/ERN for Stage‐2 errors, *t*(23) = 2.48, *p* = 0.021, d = 0.51, as well as the Ne/ERN for Stage‐1 errors, *t*(23) = 3.66, *p* < 0.001, d = 1.53 (Figure 4).

### 3.3 Multivariate Pattern Analysis

**FIGURE 5:**
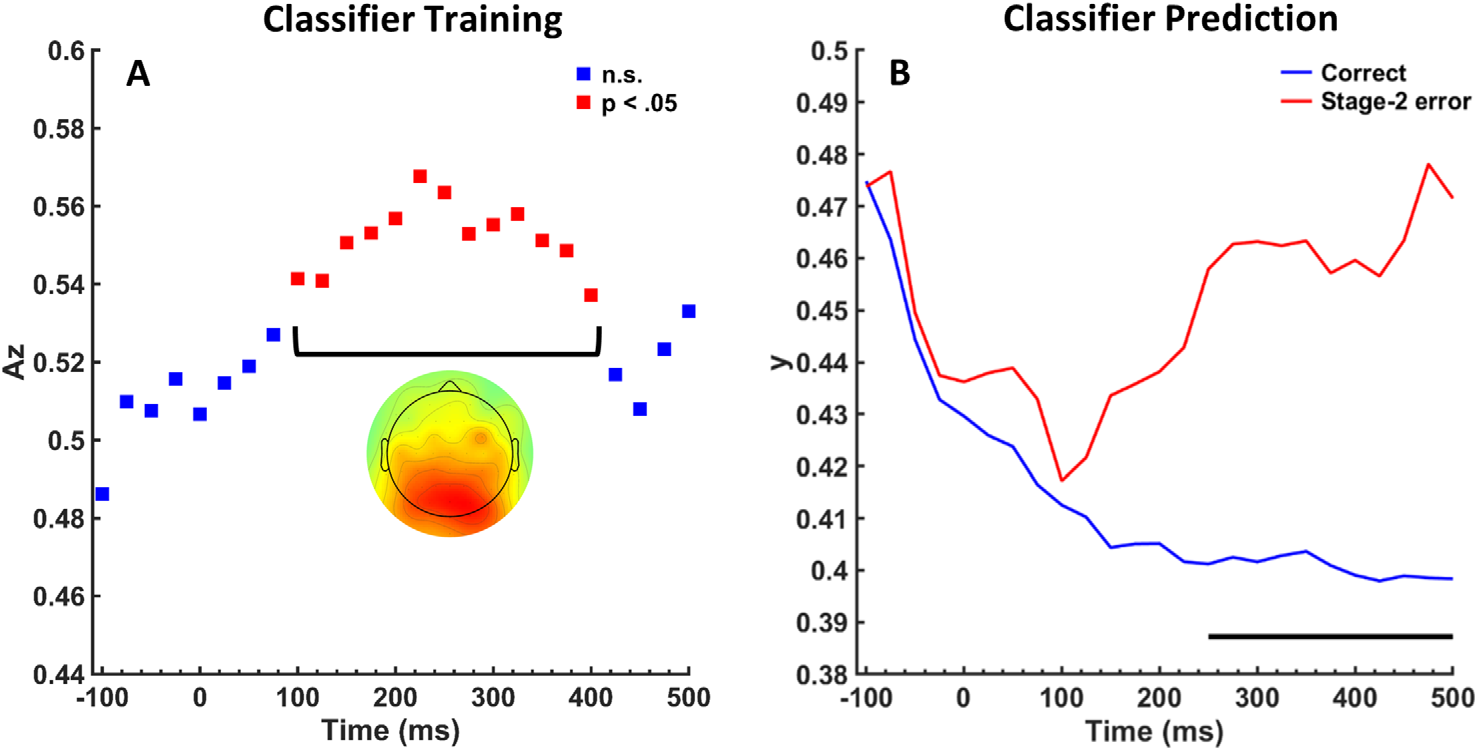
Multivariate pattern analysis. (A) Discrimination performance (Az) after training of a multivariate pattern classifier that distinguishes between correct Stage‐2 responses following Stage‐1 errors (higher‐order errors) and Stage‐2 responses following correct Stage‐1 responses. Red squares indicate time windows with a significant discrimination performance as revealed by a permutation test. The topography shows the discriminating component averaged across the significant time windows (200–500 ms). (B) Prediction values (y) after applying the classifier to Stage‐2‐errors and correct Stage‐2‐responses (both following correct Stage‐1 responses). The black bar shows the time window in which prediction values of both conditions differ significantly (*p* < 0.05).

An MVPA was used to demonstrate that the higher‐order Pe involves activity with a similar spatial distribution as the Pe on error trials, which would provide evidence that the positivity following higher‐order errors is indeed a Pe. We trained a classifier to discriminate between correct Stage‐2 trials that follow Stage‐1 errors and those that follow a correct Stage‐1 response. Az scores representing classifier performance for consecutive time windows are depicted in Figure 5A. Permutation tests revealed that classifier performance is significantly above chance for time windows between 100 and 400 ms, which overlaps with the time window of the Pe. Also, the spatial distribution of the discriminating component in this time window reveals a pattern similar to a Pe.

In a second step, we investigated whether this classifier can decode Stage‐2 errors. To this end, we used the classifier to distinguish between Stage‐2 errors and correct Stage‐2 responses (both following a correct Stage 1). This resulted in prediction values for each time window and each trial type, which are depicted in Figure 5B. We expected higher prediction values for Stage‐2 errors than for correct Stage‐1 responses if the Pe on Stage‐2 errors and the higher‐order Pe share similar spatial features. In line with this expectation, we found a significant difference in prediction values in the time window of the Pe (200–400 ms), *t*(23) = 3.03, *p* = 0.006, d = 0.618. We further tested whether this effect is specific for the Pe time window or whether a similar difference can be found for the Ne/ERN time window. In the Ne/ERN time window previously obtained for Stage‐2 errors (−25 to 25 ms), no significant difference between prediction values was observed, *t*(23) = 0.70, *p* = 0.494, d = 0.143.

Please note that the prediction values are the output of a logistic regression model. Accordingly, values above 0.5 indicate that a trial is more likely to be an error (vs. correct) while values below 0.5 indicate that a trial is more likely to be correct (vs. an error). The results in Figure 5B thus indicate that Stage‐2 errors are not identified as errors but rather as less correct trials. This could reflect that the classifier is somewhat biased towards correct trials because trials of this category were used for training (although we did not use the same correct trials for training and prediction). However, this does not change our interpretation. A classifier that is trained to decode the higher‐order Pe predicts differences between correct and Stage‐2 errors. And therefore, activity with a similar spatial distribution underlies the difference between correct and higher‐order errors (for which the classifier was trained) and the difference between correct and Stage‐2 errors (to which the classifier was applied).

## 4 Discussion

The present study investigated the idea that error monitoring evaluates the overall outcome of a multistage task beyond the outcome of individual stages, and that this overall outcome is reflected by the Pe. This assumption was tested in a two‐stage task in which the overall outcome depended on the performance at each individual stage. Crucially, if an incorrect response was provided at Stage 1, even a correct response at Stage 2 led to an unfavorable outcome because a draw is less favorable than a win as it does not result in monetary gain. Our results show that this case led to a posterior positivity after the correct Stage‐2 response, which we interpreted as a higher‐order Pe. This interpretation received support from an MVPA demonstrating that a pattern classifier trained on this higher‐order Pe could decode the Pe observed after incorrect Stage‐2 responses. In conclusion, the evidence presented here supports the idea of a higher‐order error monitoring system which evaluates the overall outcome in a multistage task, and which is reflected by a Pe.

Our results suggest that the Pe reflects more than the accumulated evidence for an error in the immediately preceding response. The evidence accumulation account (Steinhauser and Yeung 2010; Ullsperger et al. 2010) states that the Pe represents the accumulated evidence that an error has been committed. While this can explain the Pe following incorrect Stage‐1 and Stage‐2 responses as well as corresponding results from single‐stage tasks (Murphy et al. 2015; Navarro‐Cebrian et al. 2016; Steinhauser and Yeung 2010, 2012; Ullsperger et al. 2010), it is insufficient to account for the higher‐order Pe following correct Stage‐2 responses. Our results are also difficult to explain by the idea that the Pe reflects the confidence of having made an error (Boldt and Yeung 2015). Even though no confidence judgments were assessed in the present study, there is no reason to assume that participants' confidence at Stage 2 is impaired because of an incorrect Stage‐1 response, as there was enough time between stages to complete post‐decisional processing of Stage 1. We thus interpret the higher‐order Pe as reflecting the outcome of the whole multistage task. More specifically, the Pe for correct Stage‐2 responses indicates that the overall outcome of the trial is unfavorable (as it corresponds to a draw), even though the Stage‐2 response is correct (as it corresponds to the better of two options). We call this Pe a higher‐order Pe because it represents an evaluative signal that takes the whole multistage task into account rather than a single response.

The higher‐order Pe is smaller than the Pe for Stage‐1 errors and Stage‐2 errors. Relative to the pre‐response baseline, the amplitude on higher‐order errors in the Pe time window is even negative, and a positivity is obtained only because we measured the Pe as the difference between correct and higher‐order error trials. Given that only some studies in the literature use this difference approach (e.g., Boldt and Yeung 2015; Di Gregorio et al. 2016; Falkenstein et al. 1990, 1991) whereas others use the absolute amplitude for error trials (e.g., Endrass et al. 2010; Hewig et al. 2011), the question emerges as to whether the higher‐order Pe defined as a difference meets the characteristics of a true Pe. We believe that defining the Pe as a difference between correct and error trials leads to a better estimate of late error‐related brain activity because the time course of response‐locked activity on correct and error trials is strongly affected by stimulus‐locked components such as the P300. The immediate decline of posterior activity after the (correct and incorrect) response at each stage in Figures 2 and 3 suggests that the response coincides with the peak of the P300 in our data and that the decline reflects the decrease of the P300 after this peak. As a consequence, a valid quantification of the Pe is possible only if this P300 time course is subtracted by using the difference between correct and error trials. In the Supporting Information, we provide evidence for this view by analyzing epochs comprising both stages with a pre‐stimulus baseline, in which the influence of the stimulus‐locked P300 can be clearly identified. In these analyses, we also show that the higher‐order Pe as well as all other reported forms of error‐related brain activity are replicated with a pre‐stimulus baseline. This additionally indicates that our results cannot be accounted for by effects during the pre‐response period.

A further question is whether the higher‐order Pe and the Pe following an incorrect response reflect the same or different mechanisms. From the latter perspective, one might argue that the two types of Pe reflect two levels of error monitoring, the level of individual responses and the level of the multistage task. While the former takes place after each response, the latter additionally occurs at the end of a trial. The higher‐order monitoring process could integrate individual error signals from each stage with task rules specifying the overall outcome (e.g., “error at Stage 1 and correct response at Stage 2 results in a draw”) to provide evidence for an unfavorable outcome reflected by the higher‐order Pe. Such an inference‐based metacognitive process could fulfill functions such as identifying errors that are not characterized by a single incorrect response, such as order errors in dual tasking (e.g., Steinhauser et al. 2021). It could also underlie the ability to report the source of an unfavorable outcome (Di Gregorio et al. 2016). Solving the so‐called credit assignment problem (Minsky 1961), that is, the problem of assigning an outcome to its underlying source, is crucial for learning from errors and is particularly relevant in the context of multistage tasks (e.g., Fu and Anderson 2008; Stolyarova 2018; Walsh and Anderson 2014; Wurm et al. 2024).

Alternatively, the higher‐order Pe and the Pe following incorrect responses could reflect the same mechanism. It is conceivable that both represent a neural signal indicating that the current response led to an unfavorable outcome, irrespective of whether this is due to an error in a single response or a specific response pattern in a multistage task. The higher‐order Pe in our paradigm could reflect the influence of context on the evidence accumulation process. A correct Stage‐1 response leads to a win context, which implies that only an incorrect Stage‐2 response entails an unfavorable outcome. However, an error at Stage 1 leads to a loss context, and hence, even a correct Stage‐2 response is associated with an unfavorable outcome. In this case, the context itself delivers the evidence for an unfavorable outcome, thus inducing a Pe. A necessary implication of such a single‐mechanism explanation is that any Pe reflects a higher‐order monitoring system because it takes contextual information into account and thus operates on a level beyond that of individual responses.

This single‐mechanism explanation of the Pe receives support from our decoder analysis which suggests that the higher‐order Pe and Pe after incorrect responses share similar spatial features. However, another observation might speak against this view. Trials with a “Stage‐1 correct/Stage‐2 error” pattern and trials with a “Stage‐1 error/Stage‐2 correct” pattern both lead to the same outcome (i.e., a draw). However, the Pe amplitude in the former case (i.e., after Stage‐2 errors) is substantially larger than the Pe amplitude in the latter case (i.e., after correct Stage‐2 responses). This appears to be more compatible with the idea of two types of Pe: The Stage‐2 response on “Stage‐1 correct/Stage‐2 error” trials leads to both, a Pe due to the incorrect response and a higher‐order Pe, whereas the Stage‐2 response on “Stage‐1 error/Stage‐2 correct” trials leads only to a higher‐order Pe. This putatively additive pattern of the two types of Pe might imply that the two underlying monitoring processes are independent but coincide in time. Further studies are necessary to determine whether the two types of Pe are based on a single mechanism or reflect two different levels of monitoring. This could be achieved by source modeling to identify whether the two types of Pe are generated by different networks of neural generators.

The design of the present study implies that decisions at the second stage are made either in a gain context (correct responses lead to a win, incorrect responses lead to a draw) or a loss context (incorrect responses lead to loss, correct responses lead to a draw). Several previous studies contrasted conditions with a gain, loss, or neutral context (neither gain nor loss) to investigate how motivational context influences error monitoring (Endrass et al. 2010; Maruo et al. 2016; Overmeyer et al. 2023; Potts 2011). In these studies, context was manipulated by the experimenter rather than being a consequence of an own preceding decision. While motivational context showed mixed effects on the Ne/ERN, the majority of studies found no significant effects on the Pe (Maruo et al. 2016; Overmeyer et al. 2023; Potts 2011). Only a single study reported an increased Pe for loss context versus neutral context (Endrass et al. 2010). Crucially, none of these studies reported evidence that context influenced Pe activity on correct trials. This suggests that context alone cannot account for the observation of the higher‐order Pe. It appears necessary that the unfavorable outcome was caused by the participants' own incorrect response at Stage 1. This highlights an important aspect, namely that the higher‐order Pe is still a signature of performance monitoring rather than a pure index of behavioral outcome.

The present study reports a Pe that is not caused by an error in the current response (Stage 2) but by an error in the preceding response (Stage 1). A similar pattern has been observed in dual‐tasking experiments (Steinhauser and Steinhauser 2021) in which participants responded to two independent tasks whose stimuli were separated either by a short interval (300 ms) or a long interval (1200 ms). With a short interval, it was observed that the Pe for errors in the first task was reduced, but instead a Pe emerged after the correct response in the second task. This was interpreted as reflecting an adaptive mechanism that shifts the Pe away from the initially incorrect task towards the end of the entire dual‐task to prevent interference between error monitoring and task processing (Buzzell et al. 2017; Houtman and Notebaert 2013; Jentzsch and Dudschig 2009). This interpretation received further support from the finding that the Pe to the (erroneous) first and (correct) second task response correlated negatively. It is unlikely that the higher‐order Pe in the present data is due to a similar mechanism. Steinhauser and Steinhauser (2021) observed this effect only for temporally overlapping tasks but not if stimuli were separated by a long interval. In the present study, the stimuli of the two stages are separated by about 2600 ms, which is even longer than the long interval in their study. However, both studies demonstrate that a Pe can occur following a correct response, albeit for different reasons.

Remarkably, the Ne/ERN did not show the pattern of a higher‐order error monitoring signal as it only occurred immediately after incorrect responses. This is consistent with theories of the Ne/ERN such as conflict monitoring theory (Yeung et al. 2004) or reinforcement learning theory (Holroyd and Coles 2002). Conflict monitoring predicts a Ne/ERN only if a response is followed by a corrective tendency which should never occur for correct Stage‐2 responses even if they are associated with an unfavorable outcome. Reinforcement learning theory assumes that a Ne/ERN is elicited if a response indicates an outcome that is worse than expected. The scenario in which we observed a higher‐order Pe (i.e., a correct Stage‐2 response following an error at Stage 1) causes an unfavorable outcome (draw), which is not worse than expected as the Stage‐1 error leads to a losing context in which this draw is the best possible outcome. Hence, the fact that the higher‐order Pe is not accompanied by a higher‐order Ne/ERN is fully compatible with these theories. It also confirms previous findings that a Pe can occur in the absence of a Ne/ERN, and hence, that these components reflect independent systems of error monitoring (Di Gregorio et al. 2018). A less expected outcome is that the Ne/ERN at Stage 1 (but not at Stage 2) is also observable at posterior electrodes. This is not due to the choice of the pre‐response baseline as an Ne/ERN‐like negative deflection is also observable in the stimulus‐locked data with a pre‐stimulus baseline (see Supporting Information). One possibility is that this is related to the specific motor requirements in our paradigm, which could be addressed in future studies.

The present results demonstrate that a full understanding of neural signatures like the Pe cannot be achieved if error processing is exclusively investigated in single‐stage tasks like the flanker task. More complex scenarios like our multistage task can reveal aspects of error monitoring that remain unnoticed in these simple tasks. This implies that it could be valuable to turn to more ecologically valid contexts. Real‐world decision‐making often involves multistage processes where an error in one stage can influence a final outcome (e.g., medical diagnoses, financial decisions). Recently, several studies addressed error monitoring in sports (Denul et al. 2025), car driving (Zhang et al. 2015) or immersive VR environments (Arake et al. 2022). Focusing on such settings could clarify the role of the higher‐order Pe in adaptive behavior and outcome evaluation. The lack of ecological validity of single‐stage choice tasks used in laboratory settings could also be the reason why brain activity in these tasks is only weakly related to everyday behavior (e.g., Saunders et al. 2022). Focusing on more complex tasks should be accompanied by involving more representative samples. Gender imbalances in typical studies on error monitoring—and also in the present study—could impair the generalizability of results as gender has been shown to influence neural signatures of error monitoring (Fischer et al. 2016; Larson et al. 2011).

## 5 Conclusion

The present study demonstrates that a Pe can occur if a response is associated with an unfavorable outcome even if this response was correct. This suggests that the Pe reflects an inference‐based outcome evaluation that takes place at a higher level of error monitoring that either integrates multiple error signals in a multistage task or takes context into account. In contrast, the Ne/ERN appears to operate on a lower level of error monitoring that mainly evaluates the correctness of an immediately preceding response.

## Author Contributions

**Peter Löschner:** conceptualization, formal analysis, investigation, methodology, visualization, writing – original draft. **Marco Steinhauser:** conceptualization, funding acquisition, methodology, supervision, writing – review and editing.

## Conflicts of Interest

The authors declare no conflicts of interest.

## Supporting information


**Figure S1:** Error‐related brain activity for correct trials, Stage‐1 errors (with correct Stage‐2 response) and Stage‐2‐errors (with correct Stage‐1 response) at electrode FCz. (A) Stage‐1‐locked waveforms in a time window comprising both stages. (B) Stage‐2‐locked waveforms. C and D: Difference waves between error trials and correct trials. Boxplots show the distribution of response times at the respective stage.
**Figure S2:** Error‐related brain activity for correct trials, Stage‐1 errors (with correct Stage‐2 response), and Stage‐2‐errors (with correct Stage‐1 response) at electrode Pz. (A) Stage‐1‐locked waveforms in a time window comprising both stages. (B) Stage‐2‐locked waveforms. C and D: Difference waves between error trials and correct trials. Boxplots show the distribution of response times at the respective stage.
**Figure S3:** Error‐related brain activity for correct trials, Stage‐1 errors (with correct Stage‐2 response) and Stage‐2‐errors (with correct Stage‐1 response) at electrode POz. (A) Stage‐1‐locked waveforms in a time window comprising both stages. (B) Stage‐2‐locked waveforms. C and D: Difference waves between error trials and correct trials. Boxplots show the distribution of response times at the respective stage.

## Data Availability

The data that support the findings of this study are available from the corresponding author upon reasonable request.
