# Supplementary material for: Right but Still Lousy: Correct Responses With an Unfavorable Outcome Elicit an Error Positivity
Source: Psychophysiology. 2025 Sep 19;62(9):e70151. doi: 10.1111/psyp.70151 (PMC12449677; doi:10.1111/psyp.70151)
Supplement: Supplementary file 1 — Figure S1: Error‐related brain activity for correct trials, Stage‐1 errors (with correct Stage‐2 response) and Stage‐2‐errors (with correct Stage‐1 response) at electrode FCz. (A) Stage‐1‐locked waveforms in a time window comprising both stages. (B) Stage‐2‐locked waveforms. C and D: Difference waves between error trials and correct trials. Boxplots show the distribution of response times at the respective stage. Figure S2: Error‐related brain activity for correct trials, Stage‐1 errors (with correct Stage‐2 response), and Stage‐2‐errors (with correct Stage‐1 response) at electrode Pz. (A) Stage‐1‐locked waveforms in a time window comprising both stages. (B) Stage‐2‐locked waveforms. C and D: Difference waves between error trials and correct trials. Boxplots show the distribution of response times at the respective stage. Figure S3: Error‐related brain activity for correct trials, Stage‐1 errors (with correct Stage‐2 response) and Stage‐2‐errors (with correct Stage‐1 response) at electrode POz. (A) Stage‐1‐locked waveforms in a time window comprising both stages. (B) Stage‐2‐locked waveforms. C and D: Difference waves between error trials and correct trials. Boxplots show the distribution of response times at the respective stage. [file PSYP-62-e70151-s001.docx]

# **Supplementary materials**

The present study reports a higher-order Pe for correct Stage-2 responses that follow a Stage-1 error. The question emerges whether this positivity is related to any differences occurring between the two stages and thus is influenced by the pre-response baseline. To explore this issue, we present data from large stimulus-locked epochs that comprise a) both stimuli and responses, and b) only Stage-2 stimuli and Stage-2 responses, and we applied a pre-stimulus baseline to both. The goal of these analyses is, first, to investigate how the time course of stimulus-locked activity influences the calculation of the Pe, and second, to investigate whether a Pe can also be found when applying a stimulus-locked baseline.

*Data analysis.* Preprocessing was the same as for the response-locked data except for the following differences. We extracted epochs ranging from -500 ms to 5000 ms relative to the Stage-1 stimulus and epochs ranging from -500 ms before to 2000 ms relative to the Stage-2 stimulus. For each, a baseline of -200 ms to 0 ms was applied. To ensure comparability to the response-locked data, trials for artifact exclusion were not newly determined but were rather adopted from the response-locked analyses. For the epochs locked to Stage-1 stimuli, trials were excluded that were also excluded in either the Stage-1 or Stage 2 response-locked analyses. For the epochs locked to Stage-2 stimuli, trials were excluded that were also excluded in the Stage-2 response-locked analysis. Again, data were averaged for three conditions: Correct trials (correct responses at both stages), Stage-1 errors (error at Stage 1 but correct at Stage 2), and Stage-2 errors (correct at Stage 1 but error at Stage 2). The number of trials remaining after preprocessing ranged between 13.38 trials (*SE* = 2.13) for Stage-2 errors in the Stage-1-locked data and 327.83 trials (*SE* = 11.58) for correct trials in the Stage-2-locked data.

*Results.* The data are presented for the electrodes FCz (Fig. S1) and POz (Fig. S3) for which we previously analyzed the data as well as for electrode Pz (Fig. S2) at which the stimulus-locked P300 is typically maximal. The left column in each figure depicts waveforms and difference waves in a large Stage-1-locked epoch that comprises stimuli and responses for both Stage 1 and Stage 2. Boxplot show the location of both responses. Please note that the location of the Stage-2 stimulus is variable and depends on the Stage-1 response in this figure. The right column therefore depicts waveforms and difference waves for Stage-2-locked data.

Because we analyzed the Pe at electrode POz, Figure S3 provide the data relevant for this analysis. Figure S3A shows that there is a pronounced stimulus-locked P300 that peaks at around the responses at Stage 1 and Stage 2. The P300 peaks at electrode Pz (Fig. S2A) but is still large at POz. A Pe for Stage-1 errors emerges because waveforms for correct and error trials diverge right after the P300 peak (left part of Figs. S3AC). The time course of the waveforms nicely illustrates why the Pe is better characterized by the difference between corrects and errors. Because the Stage-1 response coincides with the peak of the stimulus-locked P300, the post-response period shows a sharp decline for corrects and errors. The Pe emerges as a positivity relative to this decline. Because the absolute amplitude on error trials is always more negative than the pre-response baseline, characterizing the Pe as this absolute amplitude would result in a negativity rather than a positivity. However, this is a consequence of the fact that the response coincides with the P300 peak, and a different pattern would emerge if the response occurred earlier. Using the difference between corrects and errors to quantify the Pe has the advantage that it provides a measure of the Pe that is more independent of stimulus-locked activity, at least when corrects and errors do not differ in RT as in the present data.

Figure S3BD shows that the same conclusions can be drawn for Stage-2 errors which also emerge right after the P300 peak. Crucially, it also holds for the higher-order Pe. In the figures, the Pe for higher-order errors can be seen after the (correct) Stage-2 response in the waveforms for Stage-1 errors. The Pe for higher-order errors is much smaller than that for Stage-2 errors but it also emerges right after the P300. Comparing the Figures S3A and S3B shows that the Pe for higher-order errors does not result as a carry-over effect of pre-response differences influencing the pre-response baseline. Figure S3A reveals a long-going negativity for trials with Stage-1 and Stage-2 errors between the two stages which presumably reflects a contingent negative variation (CNV) or stimulus-preceding negativity (SPN) (Brunia, 1988; Brunia & Damen, 1988), which carries over into post-response activity at Stage-2 if a baseline prior to Stage 1 is applied (as in Fig. S3A). However, if a baseline prior to Stage 2 is used (as in Fig. S3B), the waveforms of all trial types overlap and diverge not until the response. This also demonstrates that the pre-response baseline used in the main analyses is not responsible for the emergence of the higher-order Pe. However, a closer look shows that the P300 for higher-order errors is slightly smaller than that for corrects, which could have affected the pre-response baseline. We therefore re-analyzed the Pe with a pre-stimulus baseline for all error types to show it is still significant if no pre-response baseline is applied.

We conducted the analysis in the data depicted in Figure S3, and thus, in data with a baseline prior to the Stage-1 stimulus or Stage-2 stimulus. However, we calculated the Pe in the same post-response time window as in the main analyses. That is, we extracted the mean amplitude from 200 ms to 400 ms after the respective response for each trial and averaged these data for each condition. This effectively resulted in a response-locked analysis with a pre-stimulus baseline.

The Pe for Stage-1 errors was analyzed in data with a baseline prior to the Stage-1 stimulus (Fig. S3AC). Amplitudes in the Pe time window were more positive for Stage-1 errors (*M* = 7.92 µV, *SE* = 1.69 µV) than for corrects (*M* = 2.55 µV, *SE* = 1.01), *t*(23) = 3.93, *p* < .001, $d$ = 0.80. The Pe for Stage-2 errors and higher-order errors were analyzed in data with a baseline prior to the Stage-2 stimulus (Fig. S3BD). Amplitudes were more positive for Stage-2 errors (*M* = 8.56 µV, *SE* = 2.15 µV) than for corrects (*M* = 1.27 µV, *SE* = 1.01 µV), *t*(23) = 3.90, *p* < .001, $d$ = 0.80, and more positive for higher-order errors (*M* = 3.54 µV, *SE* = 1.29 µV) than for corrects, *t*(23) = 2.54, *p* = .002, $d$ = 0.52. This shows that the three types of Pe are fully replicable when using a pre-stimulus baseline.

For completeness, we also applied the analyses of the Ne/ERN to the data from electrode FCz (Fig. S1). We computed cluster-based permutation tests to the response-locked time windows with pre-stimulus baselines. Similar results as in the main analyses were obtained. For Stage-1 errors, errors were more negative than corrects in a cluster ranging from -47 ms to 105 ms (*p* < .001). For Stage-2 errors, a significant negative cluster from -39 ms to 51 ms was revealed (*p* = .005). But no significant cluster for higher-order errors was obtained.

**References**

Brunia, C. H. M. (1988). Movement and stimulus preceding negativity. *Biological Psychology*, *26*(1–3), 165–178. https://doi.org/10.1016/0301-0511(88)90018-X

Brunia, C. H. M., & Damen, E. J. P. (1988). Distribution of slow brain potentials related to motor preparation and stimulus anticipation in a time estimation task. *Electroencephalography and Clinical Neurophysiology*, *69*(3), 234–243. https://doi.org/10.1016/0013-4694(88)90132-0


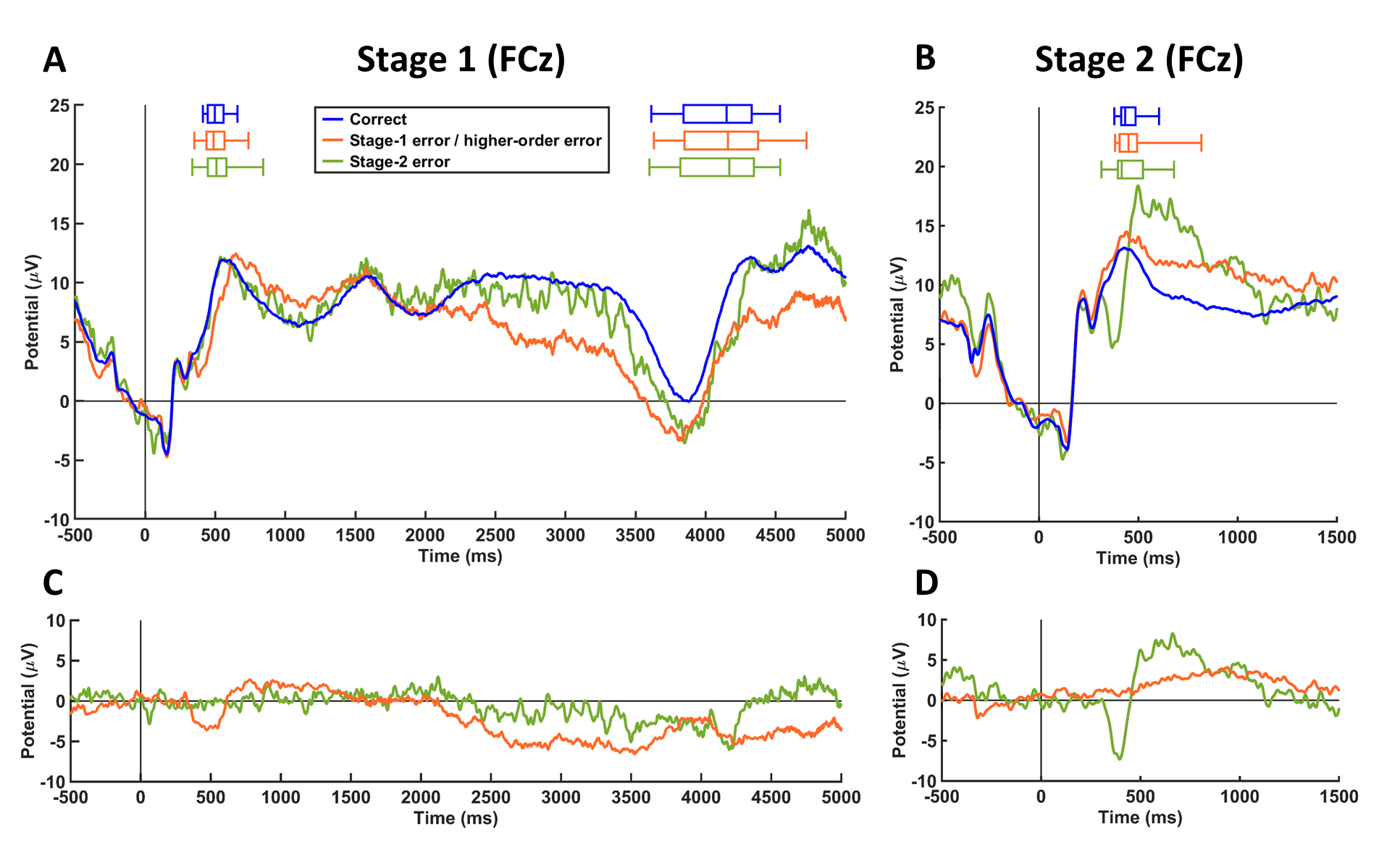
*Figure S1.* Error-related brain activity for correct trials, Stage-1 errors (with correct Stage-2 response) and Stage-2-errors (with correct Stage-1 response) at electrode FCz. A: Stage-1-locked waveforms in a time window comprising both stages. B: Stage-2-locked waveforms. C and D: Difference waves between error trials and correct trials. Boxplots show the distribution of response times at the respective stage.


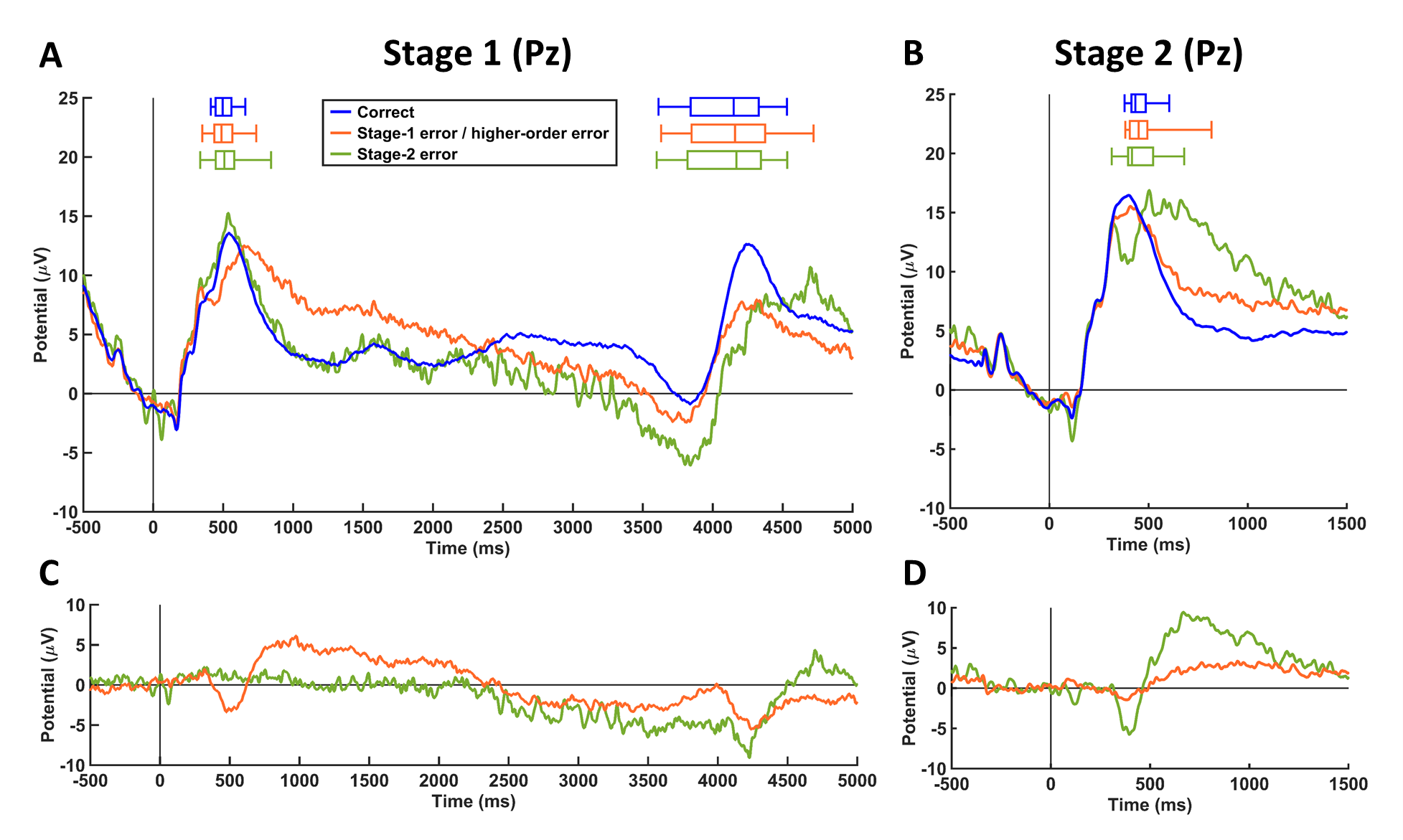
*Figure S2.* Error-related brain activity for correct trials, Stage-1 errors (with correct Stage-2 response) and Stage-2-errors (with correct Stage-1 response) at electrode Pz. A: Stage-1-locked waveforms in a time window comprising both stages. B: Stage-2-locked waveforms. C and D: Difference waves between error trials and correct trials. Boxplots show the distribution of response times at the respective stage.


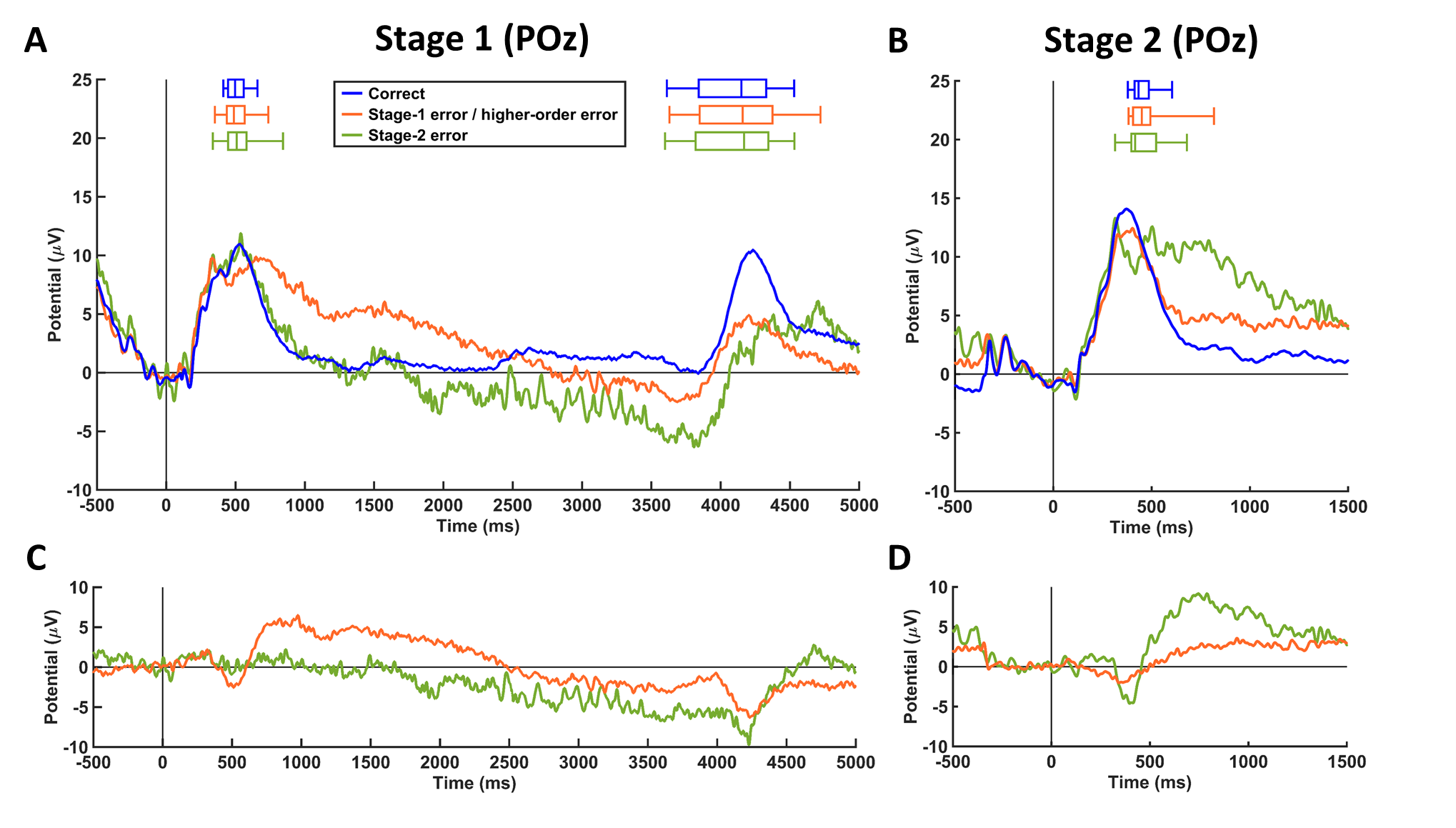
*Figure S3.* Error-related brain activity for correct trials, Stage-1 errors (with correct Stage-2 response) and Stage-2-errors (with correct Stage-1 response) at electrode POz. A: Stage-1-locked waveforms in a time window comprising both stages. B: Stage-2-locked waveforms. C and D: Difference waves between error trials and correct trials. Boxplots show the distribution of response times at the respective stage.
